# Bioassay-Guided Fractionation of Acetone and Methanol Extracts of *Quercus infectoria* Galls with Antimalarial Properties

**DOI:** 10.21315/tlsr2024.35.2.8

**Published:** 2024-07-31

**Authors:** Nurul Hammizah Hamidon, Anjana Chamilka Thuduhenage Dona, Nik Nor Imam Nik Mat Zin, Nurul Izza Nordin, Shaida Fariza Sulaiman, Nurhidanatasha Abu-Bakar

**Affiliations:** 1 School of Health Sciences, Universiti Sains Malaysia, Health Campus, 16150 Kubang Kerian, Kelantan, Malaysia; 2 SIRIM BERHAD, Institute of Biotechnology Research Centre, Block 19, No. 1, Persiaran Dato Menteri, Section 2, 40700 Shah Alam, Selangor, Malaysia; 3 School of Pharmaceutical Sciences, Universiti Sains Malaysia, 11800 USM Pulau Pinang, Malaysia

**Keywords:** *Quercus infectoria*, Antimalarial Activity, *Plasmodium falciparum*, Preparative-HPLC, HR-LCMS, *Quercus infectoria*, Aktiviti Antimalaria, *Plasmodium falciparum*, Persediaan-HPLC, HR-LCMS

## Abstract

The antimalarial properties of crude extracts from *Quercus infectoria* galls were investigated through bioassay-guided fractionation. Acetone (QIA) and methanol (QIM) crude extracts have been reported to have promising antimalarial activity against *Plasmodium falciparum* (3D7 strain). These extracts were subjected to fractionation using automated preparative high-performance liquid chromatography (prep-HPLC) to identify the most active fractions. Nine fractions were isolated from each extract, of which the fractions QIA11 and QIM16 showed antimalarial activity, with IC_50_ values of 17.65 ± 1.82 μg/mL and 24.21 ± 1.88 μg/mL, respectively. In comparison, the standard antimalarial drug artemisinin has an IC_50_ value of 0.004 ± 0.001 μg/mL). Through high-resolution liquid chromatography coupled with mass spectrometry (HR-LCMS) analysis of the fractions, four known compounds were successfully identified: gallic acid, ellagic acid, 1,3,6-tris-o-(3,4,5-trihydroxybenzoyl)-beta-d-glucose and 1-O,6-O-digalloyl-beta-D-glucose.

## Abstrak

Sifat-sifat antimalaria ekstrak-ekstrak mentah dari biji *Quercus infectoria* dikaji melalui fraksinasi berpandukan bioasai. Ekstrak mentah aseton (QIA) dan metanol (QIM) dilaporkan sebelum ini mempunyai aktiviti antimalaria terhadap *Plasmodium falciparum* (strain 3D7). Ekstrak-ekstrak ini melalui fraksinasi menggunakan persediaan automatik kromatografi cecair berprestasi tinggi (prep-HPLC) untuk mengenal pasti fraksi yang paling aktif. Sembilan fraksi telah diasingkan daripada setiap ekstrak, di mana fraksi QIA11 dan QIM16 menunjukkan aktiviti antimalaria dengan nilai IC_50_ 17.65 ± 1.82 μg / mL dan 24.21 ± 1.88 μg/mL, masing-masing dibandingkan dengan ubat antimalaria standard, artemisinin (IC_50_ = 0.004 ± 0.001 μg/mL). Analisa menggunakan kromatografi cecair beresolusi tinggi bersama dengan spektrometri jisim (HR-LCMS) berjaya mengenal pasti empat sebatian: asid gallic, asid ellagic, 1,3,6-tris-o-(3,4,5-trihydroxybenzoyl)-beta-dglukosa dan 1-O, 6-O-digalloyl-beta-D-glukosa.

**Highlights**

- Acetone (QIA) and methanol (QIM) crude extracts were reported to have promising antimalarial activity against *Plasmodium falciparum* (3D7 strain).
- The antimalarial properties of crude extracts from *Quercus infectoria* galls were investigated through bioassay-guided fractionation and identification of compounds through high-resolution liquid chromatography coupled with mass spectrometry (HR-LCMS) analysis.
- Four known compounds were successfully identified which was isolated from two most active fractions, fractions QIA11 and QIM16.

## INTRODUCTION

Malaria is a parasitic disease that has a negative impact on global health (Ferguson 2018). In many developing countries, the disease causes major complications leading to high morbidity and mortality (Nigussie & Wale 2022). The five *Plasmodium* species responsible for human malaria are *P. falciparum, P. vivax, P. ovale, P. malariae* and *P. knowlesi* (Nigussie & Wale 2022). *P. falciparum* is the most threatening species in terms of morbidity and mortality (Ouji *et al*. 2018). According to the World Health Organisation (WHO), an estimated 247 million cases of malaria occurred worldwide in 2021, compared with 245 million cases in 2020 (WHO 2021), which resulted in 619,000 deaths. The WHO African Region accounted for the majority of malaria cases (95%) and deaths (96%), with children under five years of age (80%) being those mainly affected by the disease (WHO 2021).

Plants are an excellent source of novel natural products. Medicinal plants have long been used as a source of therapeutic agents and they have demonstrated beneficial uses in a variety of applications (Yuan *e t al*. 2016). Despite intense competition from synthetic compounds, numerous bioactive substances found in plants have been shown to be significantly important in advancing human health (Tahir *et al*. 2022). Between 1981 and 2014, the Food and Drug Administration (FDA) approved 1,562 drugs in the United States, of which 4% were unaltered natural products, 9% were botanical drugs, 21% were natural derivatives and 4% were synthetic drugs containing natural pharmacophores (Newman & Cragg 2020). These findings have increased the interest in herbal medicine (Che & Zhang 2019).

There are numerous examples of effective contemporary drugs based on medicinal plants, especially with the discovery of new drug candidates for the treatment of many infectious diseases like malaria (Nakalembe *et al*. 2019; Atanasov *et al*. 2015). Medicinal plants have enormous potential for use in the effective management of various strains of malaria parasites, including those resistant to the available antimalarial drugs (Shah *et al*. 2014). In the absence of viable malaria vaccinations, accurate diagnosis and treatment remain the best hope of avoiding serious consequences. Several antimalarial medications have been discovered for this purpose, including mefloquine, chloroquine, quinine, proguanil, atovaquone, sulfadoxine-pyrimethamine and artemisinin (Arya *et al*. 2021). Artemisinin, which was discovered by Tu Youyou in the 1970s, originally came from the plant *Artemisia annua*, which was commonly used in Chinese medicine (Tu 2011). Artemisinin-based combination therapies (ACTs) are recognised for their effectiveness to swiftly reduce the number of *Plasmodium* parasites in the blood of patients with malaria (Ouji *et al*. 2018). However, *P. falciparum* resistance to ACTs has emerged, posing a threat to the global elimination of malaria. Due to this phenomenon, the development of new antimalarial drugs especially derived from medicinal plants is urgently needed (Shah *et al*. 2014).

*Quercus infectoria*, commonly referred to as the gall oak tree, is a small shrub 4–6 feet tall which originates mainly from Greece, Asia Minor and Iran (Abdul Haque *et al*. 2016). The galls of the plant are formed when the wasp species *Adleria gallae-tinctoria* or *Cynips gallae-tinctoria* deposit their eggs on the branches of young trees. The subsequent enzymatic reaction results in the appearance of hard galls (Wan Nor Amilah *et al*. 2022). These globular-shaped galls, known as *majuphal* or *machakai* in India and *manjakani* in Indonesia and Malaysia (Samuelsson 1999; Fatima *et al*. 2001), are 0.8 cm–2.5 cm in diameter and hard in consistency. They have a rough surface and a greyish-brown to brownish-black colour (Shaikh Imtiyaz *et al*. 2013).

Traditional uses of *Q. infectoria* galls have prompted researchers to investigate and validate their biological activities and therapeutic uses. Extracts, fractions and single compounds of the galls have been shown to have various pharmacological activities, including antioxidant, anti-inflammatory, antitumoural, antibacterial, antiviral, antifungal and antimalarial activities (Wan Nor Amilah *et al*. 2022; Morales 2021; Iylia Arina & Harisun 2019; Basri *et al*. 2012; Nik Mat Zin *et al*. 2019a; 2019b). The phytochemicals of the *Q. infectoria* galls highlight the abundance of phenolic compounds belonging to the pyrogallol, quercetin, tannins, gallic acid and ellagic acid classes (Kheirandish *et al*. 2016; Dash *et al*. 2016; Tayel *et al*. 2018; Ma *et al*. 2020; Kamarudin, Nik Salleh, *et al*. 2021; Kamarudin, Muhamad, *et al*. 2021). The phenolic compounds of the galls have been hypothesised to have antimalarial effects on haemoglobin degradation and haem detoxification in the digestive vacuole of *P. falciparum* (Tajuddeen & Van Heerden 2019; Mamede *et al*. 2020).

The preliminary study revealed that aqueous, ethanol, methanol and acetone crude extracts from *Q. infectoria* galls had antimalarial activity against the chloroquine-sensitive strain (3D7) of *P. falciparum*, with IC_50_ values of 30.95, 20.00, 10.31 and 5.85 μg/mL, respectively (Nik Mat Zin *et al*. 2020). These extracts were non-toxic to normal kidney epithelial cells (Vero) and mildly toxic to normal embryo fibroblast cells (NIH/3T3). According to Berthi *et al*. (2019) and Jonville *et al*. (2008), the antimalarial activity of an extract can be considered very active with an IC_50_ < 5 μg/mL, promising with an IC_50_ of 6 μg/mL–15 μg/mL, moderate with an IC_50_ of 16 μg/mL–30 μg/mL, low with an IC_50_ of 31 μg/mL–50 μg/mL, and inactive if the IC_50_ > 50 μg/mL. Thus, the antimalarial activity of the acetone and methanol extracts in this study was classified as promising. Here, bioassay-guided fractionation was used to identify potential active compounds in the acetone and methanol crude extracts of *Q. infectoria* galls, as well as evaluate the antimalarial activity of their fractions.

## MATERIALS AND METHODS

### Chemicals and Reagents

Acetonitrile, acetone, methanol, formic acid and trifluoroacetic acid were HPLC-grade with 99% purity and purchased from Fisher Scientific (Malaysia) Limited. Ultrapure water with 18.2 Ώm was used for gradient elution in the prep-HPLC and HR-LCMS.

### Plant Material and Extraction Procedure

*Q. infectoria* galls were purchased from a local market in Kota Bharu, Kelantan, Malaysia and authenticated at the Natural Medicinal and Product Centre, International Islamic University Malaysia (voucher specimen: PIIUM 0229-1). The galls were washed and dried at 50°C before being ground to obtain a powder. The acetone crude extract, which exhibited the highest antimalarial activity (Nik Mat Zin *et al*. 2020), was prepared by soaking 100 g of the powdered material in 500 mL of 100% acetone. The maceration technique was used at room temperature for 72 h and the extracts were filtered and concentrated using a rotary evaporator. The same process was followed to prepare the methanol crude extract using methanol (100%).

### Fractionation of Gall Crude Extracts

Fractionation was achieved using prep-HPLC (GX-281 Purification System) combined with a Supelco RP-C18 preparative column (10 mm × 250 mm, 5 μm particle size, Merck, Germany). The extracts were dissolved in either acetone or methanol (100%) to achieve a concentration of 100 mg/mL. Subsequently, 100 μL of this solution was injected into the liquid chromatography. Then, 0.0085% trifluoroacetic acid (TFA) in deionised water (A) and acetonitrile (B) was used as the mobile phase. Peaks were detected at a wavelength of 254 nm and eluted with a gradient system mixture for 36 minutes. The gradient elution procedure was as follows: B was increased from 0% to 5% at 0 to 5 min and B was increased from 5% to 95% at 5 to 27 min. Next, B was flowed at 95% for 3 min and B was decreased from 95% to 5% for 6 min at a flow rate of 2.8 mL/min. The chromatographed fractions of acetone and methanol were collected individually and vacuum-dried below 45°C. The fractions were subjected to analysis of the antimalarial activity and compound identification using HR-LCMS (Harborne 1998).

### Culture of the Malaria Parasite

A chloroquine-sensitive strain (3D7) of *P. falciparum* was kindly provided by the Institute for Research in Molecular Medicine (INFORMM), Health Campus, Universiti Sains Malaysia (USM). It was maintained in culture flasks containing a complete culture medium (CCM) and washed type O^+^ human erythrocytes at 2% haematocrit, based on a previously devised protocol (Mohd-Zamri *et al*. 2017). Human blood was acquired from healthy donors who gave informed consent and were recruited at the School of Health Sciences, Health Campus, USM. The nature and risks of the study were approved by the Human Research Ethics Committee, USM (USM/JEPeM/18050263). Donors were notified of this before being recruited.

### Synchronisation of the Malaria Parasite

The parasites were mainly at the ring stage (2% parasitaemia) upon confirmation by Giemsa-stained thin blood smears. They were synchronised through sorbitol treatment at a ratio of 100 μL of cell pellets per 1,000 μL of 5% D-sorbitol (w/v; Sigma Aldrich, Missouri, USA) to kill the mature-stage parasites (trophozoite and schizont stages) (Ibrahim & Abu-Bakar 2019). Synchronised ring-stage parasite-infected erythrocytes (2 h post-synchronisation) were used in the antimalarial activity assay.

### Malarial SYBR Green I Fluorescence-based (MSF) Assay

The antimalarial activity of the acetone and methanol fractions was assessed through MSF assay using a previous method (Mohd-Zamri *et al*. 2017). Stock solutions of each fraction were diluted in the complete culture medium (CCM) at ten concentrations of two-fold dilutions into 96-well microtitre plates, and 20 μL aliquots of the fraction concentrations were transferred into individual wells in other plates containing 180 μL suspensions of synchronised ring-stage parasite-infected erythrocytes (2% parasitaemia, 2% haematocrit). Artemisinin (Sigma Aldrich, Missouri, USA) was used as a standard control, infected erythrocytes devoid of the fractions were used as a negative control and 100% DMSO was used as a positive control. Parasite plates were incubated for 48 h at 37°C in 5% CO_2_ incubator. After incubation, 180 μL aliquots of the cell suspensions were dispensed into new plates containing 20 μL solutions of 20× SYBR Green I (Invitrogen, Waltham, Massachusetts, USA), wrapped in aluminium foils and incubated for 1 hour at room temperature (Nik Mat Zin *et al*. 2019a; 2019b). The total fluorescence (TF) signal was measured with a microplate reader at the excitation (490 nm) and emission (530 nm) wavelengths. The percentage of parasite inhibition of each concentration was calculated as follows:

The mean of three half-maximal inhibitory concentration (IC_50_) values of the fractions was determined using probit regression analysis with GraphPad Prism software (Version 9).

### LC-MS/MS Analysis of the Fractions

Analysis of the compounds present in the fractions with good antimalarial activity was performed using Dionex Ultimate 3000 RS UPLC with a Thermo Scientific Q Exactive Orbitrap Hybrid Tandem Mass Spectrometer. The column used was HSS XSelect Waters C18 (4.6 mm × 250 mm, 5 μm). The gradient was linear with water (A) and acetonitrile (B), both of which were buffered with 0.1% formic acid, starting at 5% B and equilibrated for 5 min. The gradient was increased to 95% after 30 min and held for 5 min. The gradient was equilibrated for 5 min before the next injection. The flow rate of 0.8 mL/min and the column temperature of 35°C were adjusted. The sample injection used was 3 μL. The mass detection was performed in both positive and negative atmospheric pressure ionisation-electrospray source modes. The drying gas temperature (250°C), gas flow (11 L/min), nebuliser pressure (110 psig), nebuliser assistant gas temperature (350°C), capillary voltage (400 V) and collision energy (30 eV) were set. Compound Discoverer 3.1 software was used to analyse the known and unknown compounds. The software was equipped with known online libraries (mzCloud and ChemSpider) and mzLogic algorithm was applied to rank the ChemSpider results.

### Statistical Analysis

The dose-response curves from the experimental data were analysed using the GraphPad Prism 9 software package for Windows (San Diego, California USA). The data were further analysed using one-way ANOVA using the same software, with a significant difference at *p* < 0.05. Data from three different experiments were reported as mean and standard deviation (SD) values undertaken in triplicate.

## RESULTS AND DISCUSSION

### The Yield of Fractionated Extracts

The methanol crude extract produced the highest yield of dry powder (51.64%, w/v) from 100 g of the *Q. infectoria* gall powder, followed by the acetone crude extract (50.85%, w/v). The yields produced from these extracts were similar (*P* > 0.05) because their polarity indexes of methanol and acetone were the same (*P’* = 5.1). The extraction of active chemical compounds depends greatly on the solvent’s polarity, mainly because polar molecules are easily extracted using polar solvents (Goli *et al*. 2005). Therefore, the solvent used for bioactive chemical extraction must be strategically chosen because it will affect the quantity and quality of the final extract (Zhang *et al*. 2018)

**Figure 1:**
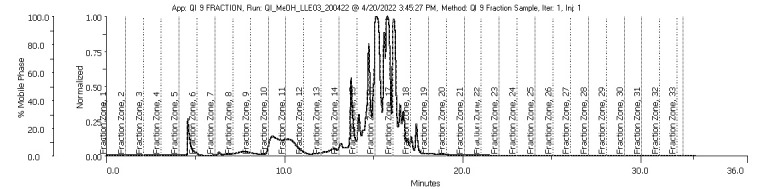
The prep-HPLC chromatogram of the methanol extract at a wavelength of 254 nm.

The crude extracts were then fractionated through a semi-preparative HPLC C18 column eluted with acetonitrile at gradient concentrations ranging from 5%–95%. The fractionation yield of the methanol and acetone extracts is shown in Table 1. The fractions QIA11 (58.88%, w/v) and QIM15 (29.80%, w/v) demonstrated the highest yield percentages for the acetone and methanol extracts, respectively. QIA11 had the highest yield due to the chromatographic co-elution between the peaks, as shown in Fig. 1. This was caused by closely eluting peaks of two or more compounds in the same fraction that were not chromatographically separated (Dworkin 2011). Peak resolution can be improved by optimising the method to increase the selectivity and efficiency of the chromatography (Vink 1972). The parameters that can be set are the chemistry of the mobile phase, the stationary phase, the temperature and the column particle size (Kanu 2021). Applying detection techniques such as mass spectrometry is the best way to distinguish co-eluting compounds that cannot be resolved through this analysis, as explained in Section 3.4 (Alseekh *et al. 2021*).

### Prep-HPLC Analysis of the Gall Crude Extracts

**Figure 2:**
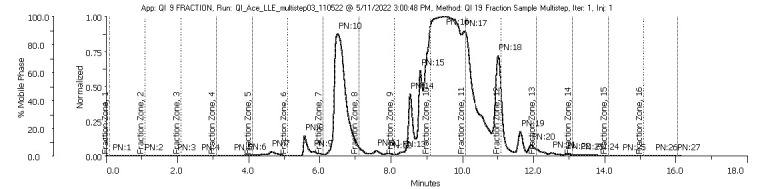
The prep-HPLC chromatogram of the acetone extract at a wavelength of 254 nm.

Both methanol and acetone crude extracts underwent the fractionation process for the isolation and semi-purification of the extracts. Fractions were collected every 1 min based on the elution of the peaks at UV absorbance values of 210 nm and 254 nm. In total, 33 fractions were collected from the methanol extract, but only nine fractions proceeded for analysis of the antimalarial activity and compound identification (as shown in Fig. 1). As for the acetone extract, 18 fractions were collected, but only nine fractions were subjected to further analysis (as shown in Fig. 2). The peaks eluted in the chromatograms represent the nine fractions of the acetone and methanol extracts, respectively. Compounds present in the fractions with chromophores that adsorbed UV at wavelengths of 210 nm and 254 nm were investigated (Shukla *et al*. 2017; Walker 2009). The chromophore is a conjugated pi-electron system that absorbs light in the region of 200 nm–800 nm (Joshi 2012); thus, compounds or molecules analysed using UV must contain pi-bonds (Shukla *et al*. 2017; Joshi 2012) to perform peaks.

Some peaks were fully eluted with good separation in fraction 06 for the methanol extract (Fig. 1) and fraction 08 for the acetone extract (Fig. 2). Although prep-HPLC is widely used for separation and detection in many applications (Kanu 2021), when it comes to complexity, plant extracts like *Q. infectoria* gall extracts are considered some of the most complex matrices. Similar to detecting pesticide residues, carbendazim and carbaryl in several paprika samples would be difficult to detect due to the interference of other compounds like indimethoate, carbofuran, imidacloprid, methomyl, spinosad and methamidophos (Ferrer Amate *et al*. 2010). The more complex the sample, the greater the challenge of developing chromatographic strategies to obtain isolated molecules (Oldoni *et al*. 2021). Sample complexity means the number of compounds in a sample is abundant; thus, the separation of sample components becomes progressively challenging. The probability of successfully separating complex samples can be improved by altering the pack capacity, which means changing the column dimension, particle size and flow rate (Snyder & Dolan 2007). This indicates that additional isolation using various techniques or different chromatographic methods (Kanu 2021; Snyder & Dolan 2007) is required to successfully achieve good separation for fractions of *Q. infectoria*.

### Antimalarial Activity of the Fractions

**Figure 3:**
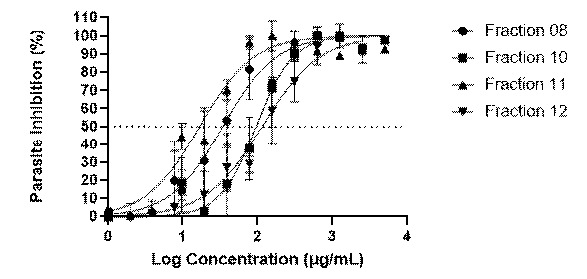

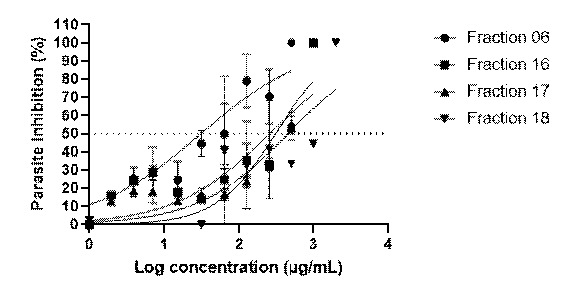

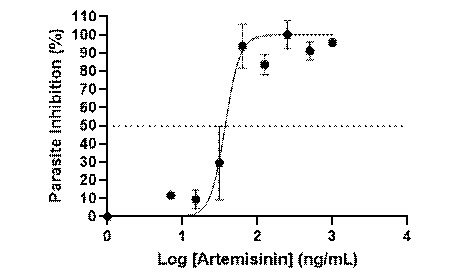
Log concentration-response curve of the: (A) acetone fractions; (B) methanol fractions; and (C) artemisinin against the chloroquine-sensitive (3D7) strain of *P. falciparum*. The horizontal dashed line corresponds to the approximate mean IC_50_ value from three independent experiments done in triplicates.

Malarial SYBR Green I fluorescence-based (MSF) assay was conducted to determine the antimalarial activity of the fractions in terms of the value of half-maximal inhibitory concentration (IC_50_). In our previous study, we revealed that acetone (IC_50_ = 5.85 ± 1.64 μg/mL) and methanol extracts (IC_50_ = 10.31 ± 1.90 μg/mL) of *Q. infectoria* galls were active against the 3D7 parasite (Nik Mat Zin *et al*. 2020). Fractionation was then performed on these extracts, followed by the *in vitro* antimalarial activity of the fractions against the parasite. For both extracts, only four of the nine fractions were analysed (Table 2). Fractions with IC_50_ values greater than 100 μg/mL were not statistically analysed as there was no antimalarial activity. When tested as a control, the artemisinin had an IC_50_ value of 0.004 ± 0.001 μg/mL (Fig. 3C). The methanol fraction QIM16 (IC_50_ = 24.21 ± 1.88 μg/mL) and acetone fraction QIA11 (IC_50_ = 17.65 ± 1.82 μg/mL) exhibited the highest antimalarial activity when compared to other fractions, as shown in Figs. 3A and 3B. The antimalarial activity of the acetone and methanol fractions was, however, lower than that of the acetone (IC_50_ = 5.85 ± 1.64 μg/mL) and methanol extracts (IC_50_ = 10.31 ± 1.90 μg/mL) reported previously (Nik Mat Zin *et al*. 2020). This finding aligns with the result reported by Ekasari *et al*. (2022), whereby 96% ethanol extract (IC_50_ = 1.88 μg/mL) from the leaves of *Sauropus androgynous* showed higher *in vitro* antimalarial activity against *P. falciparum* compared to its fraction (IC_50_ = 2.042 μg/mL). The reduction in antimalarial activity in the fractions compared to the crude extracts was also demonstrated by Ochieng *et al*. (2010), who reported that crude extracts from the aerial part of *Gardenia ternifolia* exhibited potent *in vitro* antimalarial activity against *P. falciparum*, compared to their fractions and pure isolates. The potent *in vitro* activity of the *G. ternifolia* crude extracts against the malaria parasite was possibly due to the synergistic effects of the flavonoid components (Ochieng *et al*. 2010).

To our knowledge, these are new findings regarding the comparison of the antimalarial activity of the *Q. infectoria* gall extracts and fractions, with the extracts displaying more promising antimalarial activity than their fractions against *P.falciparum*. The extracts and fractions of the *Q. infectoria* galls exhibited antimalarial activity, which could be attributed to the presence of various secondary metabolites, particularly phenolic compounds such as pyrogallol, ellagic acid, gallic acid, tannins and quercetin (Hamid *et al*. 2005; Shrestha *et al*. 2014; Tayel *et al*. 2018). The antimalarial action of these secondary metabolites has been demonstrated in their ability to inhibit the breakdown of haemoglobin and detoxify haem in the digestive vacuole of *P. falciparum* (Tajuddeen & Van Heerden 2019; Mamede *et al*. 2020). Bioactive chemicals such as phenolic compounds have been proven to suppress bacteria by disrupting cellular membranes, resulting in the loss of cellular components and finally, death. It is conceivable due to the presence of the hydroxyl (-OH) group in phenolic compounds, which has been associated with antimicrobial properties including antimalarial activity (Kumar & Goel 2019; Othman *et al*. 2019; Tajuddeen & Van Heerden 2019). The reduction in the antimalarial activity of the fractions compared to the crude extracts might be affected by the loss of synergistic activity between different compounds in the fractions (Rasoanaivo *et al*. 2021). Some compounds are inactive on their own but may act synergistically with other constituents. Comparing QIA11 and QIM16 to all other fractions, it shows that these fractions had the most compelling antimalarial activity since they isolated various phytochemical constituents. The same mechanism is applied to the *Q. infectoria* gall extracts. However, the differences in antimalarial activity might be attributed to variations in the amounts of secondary metabolites (Mazid *et al*. 2011). This indicates that the gall extracts possess a higher abundance or quality of effective antimalarial phytochemicals as compared with the fractions. Thus, antimalarial activity typically declines or ceases when the compounds become separated. In this case, the crude extracts were far more active than their fractions (Rasoanaivo *et al*. 2021). In order to further the investigation into the antimalarial properties of these active extracts, it is recommended to design an *in vivo* study using *Q. infectoria* gall extracts. This study will expedite and enhance the creation of more effective antimalarial drugs. By employing high-throughput and high-content *in-vivo* research, it is possible to accelerate the identification of new compounds, thus significantly expediting the discovery of novel antimalarial drugs.

### Identification of Chemical Constituents from the Fractions Using HR-LCMS

The different magnitudes of the antimalarial effects of the extracts and fractions may be affected by varying phytochemical compositions (Uzor *et al*. 2021). Thus, HR-LCMS analysis was run for the most prominent fractions, QIM16 and QIA11. The analysis was conducted in positive and negative modes, but only compounds with negative ions were detected. Adding 0.1% formic acid in the mobile phase often leads to formate adducts in negative mode and produces ion suppression when HR-LCMS methods are developed, especially during analysis in negative-ionisation mode (Núñez & Lucci 2014). This occurs as a result of the electrospray ionisation (ESI) mechanism in the HR-LCMS, which was affected by the excess negative charge formed either by the reduction of the mobile phase used in the analysis (i.e., water and acetonitrile) or by the presence of a weak acid (i.e., 0.1% formic acid) in the mobile phase (Kebarle & Verkerk 2010). As an acidic modifier, 0.1% formic acid provides additional protons that facilitate reduction, making it simpler for the spray droplets to carry excess negative charge (Rayleigh 1882). This negative charge excess likely accumulates on the surface of the droplet as a result of electric repulsion during negative ion ESI, increasing the pH on the surface of the droplet and creating a local environment in which deprotonation of the analytes occurs more readily than in the bulk solution (Blades *et al*. 1991). They can be strong and reproducible, and they can fragment well in MS analysis (Wu *et al*. 2004). Tables 3 and 4 show the major compounds identified in the fractions, in which the phenolic groups of gallotannin, simple phenols and tannins were identified.

Gallic acid (**1**) and 1,3,6-tris-o-(3,4,5-trihydroxybenzoyl)-beta-d-glucose (**3**) were the major compounds identified in both the methanol and acetone fractions. Gallic acid was detected at a retention time of 10.15 min with the mass found at *m/z* 171.9927. Three fragment ions were obtained in a negative mode [M-H]^−^, 68.95251, 115.05408 and 169.07555, confirming the availability of gallic acid in the fractions QIM16 and QIA11. Meanwhile, 1,3,6-tris-o-(3,4,5-trihydroxybenzoyl)-beta-d-glucose, a tannin compound in the form of galloyl glucose, was detected at 13.34 min at *m/z* 635.0865. In line with the results obtained in both this study and the previous study (Kamarudin, Nik Salleh, *et al*. 2021; Kamarudin, Muhamad, *et al*. 2021), tannin and gallotannin were identified in the *Q. infectoria* gall crude methanol and aqueous extracts using HPLC. Gallotannin, also known as tannic acid, is in the hydrolysable tannins category. It consists of a central glucose molecule esterified by gallic acid units. Although the gallotannin content in the *Q. infectoria* aqueous extract (72.0 μg/mL) was higher than in the methanol extract (46.8 μg/mL) of the same plant ((Kamarudin, Nik Salleh, *et al*. 2021; Kamarudin, Muhamad, *et al*. 2021; Abdullah *et al*. 2017), the compound was still identified in both the acetone and methanol fractions in this study.

The peak at *m/z* 483.0761 in the fraction QIA11 was 1-O,6-O-digalloyl-beta-D-glucose (**4**). This trigalloyl glucoside was also identified in negative mode and eluted at 11.71 min. Its availability was confirmed by the MS/MS spectrum ion fragmentation of 271.04473, 169.01276 and 211.02335. This compound is another tannin compound in the form of galloyl glucose that was isolated from the fraction QIA11 after 1,3,6-tris-o-(3,4,5-trihydroxybenzoyl)-beta-d-glucose (**3**).

Another compound abundant in *Q. infectoria* galls is ellagic acid (**2**). However, ellagic acid (C_14_H_6_O_8_) was only detected in the methanol fraction QIM16. The [M-H]^−^ ion of this compound was identified at *m/z* 300.9989 with a retention time of 14.78 min. The MS/MS spectrum of the peaks gave the characteristic fragments at *m/z* 125.02329, 169.01332 and 107.01264. A study by Abdullah *et al*. (2017) proved that ellagic acid was detected in a semi-purified fraction with 6.22%, a similar finding to our study on fractionation. In a review by Tajudeen and Van Heerden (2019), ellagic acid was the most active metabolite isolated from *Anogeissus leiocarpus* Combretaceae) methanol bark extracts, and it exhibited antiplasmodial activity against the 3D7 parasite, with an IC_50_ value of 18.8 μg/mL. The full HR-LCMS analysis of the fractions QIM16 and QIA11 revealed the presence of four major compounds, gallic acid, 1,3,6-tris-o-(3,4,5-trihydroxybenzoyl)-betad-glucose, 1-O,6-O-digalloyl-beta-D-glucose and ellagic acid, which could have potency as new antimalarial drug candidates. Nevertheless, further purification is needed to isolate pure compounds from the QIM16 and QIA11 fractions that exhibit higher antimalarial activity than other fractions. Purification may result in the discovery of other pure compounds in *Q. infectoria* gall with higher antimalarial activity than these semi-fractions.

## CONCLUSION

The bioassay-guided fractionation of the *Q. infectoria* gall methanol and acetone crude extracts resulted in the isolation of two active fractions QIM16 and QIA11 with varying degrees of antimalarial activity against the 3D7 strain of *P. falciparum*. The study further revealed the presence of four major compounds, identified as gallic acid (**1**), ellagic acid (**2**), 1,3,6-tris-o-(3,4,5-trihydroxybenzoyl)-beta-d-glucose (**3**) and 1-O,6-O-digalloyl-beta-D-glucose (**4**). The findings of this study confirm the ethnobotanical use of *Q. infectoria* galls as a herbal treatment for malaria, which may lead to further research on the purification of *Q. infectoria* fractions or compounds.

## Figures and Tables

**Table 1:** The yield (w/v, %) of the fractions of the *Q. infectoria* gall crude extracts.

*Q. infectoria* gall extract	Fraction	Yield (w/v,%)
Methanol (QIM)	06	11.31
	07	1.81
	11	20.49
	12	5.82
	15	29.80
	16	9.24
	17	2.84
	18	3.68
	19	15.00
Acetone (QIA)	06	2.91
	07	4.01
	08	1.19
	09	6.35
	10	10.17
	11	58.88
	12	9.86
	13	4.12
	14	2.51

**Table 2:** The antimalarial activity of the fractions of the *Q. infectoria* gall extracts.

*Q. infectoria* gall extract/drug	Fraction	IC_50_ (μg/mL)	*P*-value	F (DFn, DFd)
Methanol (QIM)	06	70.71	< 0.0001	F (8, 18) = 32.94
	07	> 100*	-	
	11	> 100*	-	
	12	> 100*	-	
	15	> 100*	-	
	16	24.21	0.0100	
	17	36.74	0.0002	
	18	54.63	< 0.0001	
	19	> 100*	-	

Acetone (QIA)	06	> 100*	-	
	07	> 100*	-	
	08	24.55	0.0089	
	09	> 100*	-	
	10	77.95	< 0.0001	
	11	17.65	0.0786	
	12	60.48	< 0.0001	
	13	> 100*	-	
	14	> 100*	-	
Artemisinin		0.004	0.001	

*Notes:*

* Data with IC_50_ values more than 100 μg/mL do not possess antimalarial activity.

The data were expressed as mean (SD) of three independent experiments. Mean values were tested for normality before proceeding to the parametric test; one-way ANOVA followed by Dunnett’s multiple comparisons at 95% confidence. Value of *P* < 0.05 was statistically significant. All methanol and acetone fractions were known as treated groups. Artemisinin was known as a control group. All fractions were statistically significant except for QIA11 was not significant with comparison of artemisinin (*P* = 0.078). Fractions with IC_50_ values more than 100 μg/mL were not statistically analysed as there were no antimalarial activity. DFn = degree of freedom numerator; DFd = degree of freedom denominator.

**Table 3:** Compounds identified in the methanol fraction QIM16 by HR-LCMS.

Compound name	Molecular structure	Retention time (min)	Found mass (*m/z*)	*m/z* values for fragment ions	Ionisation mode
Gallic acid (C_7_H_6_O_5_)	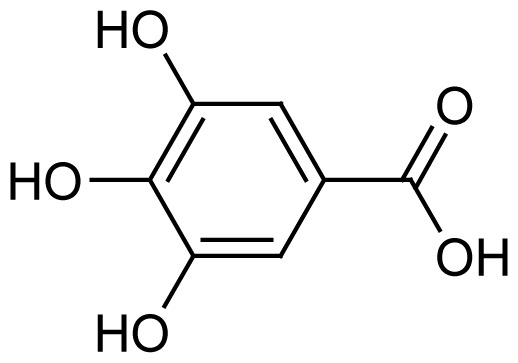 1	10.15	171.9927	68.95251, 115.05408, 169.07555	Negative
Ellagic acid (C_14_H_6_O_8_)	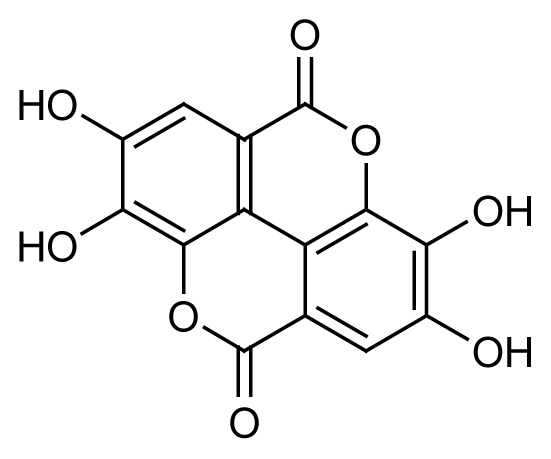 2	14.78	300.9989	125.02329, 169.01332, 107.01264	Negative
1,3,6-tris-o-(3,4,5-trihydroxybenzoyl)-beta-d-glucose (C_27_H_24_O_18_)	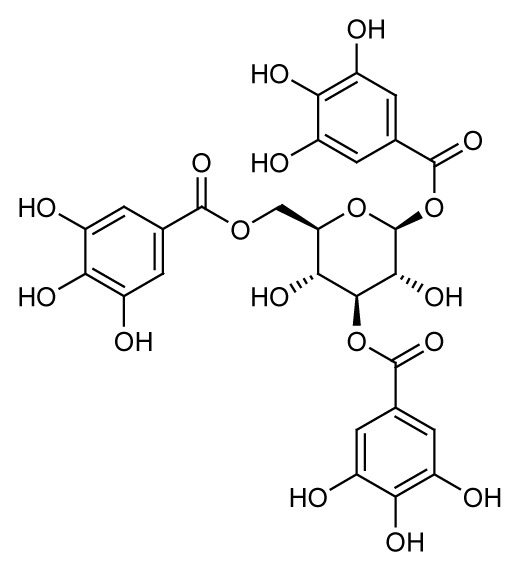 3	13.34	635.0865	483.07592, 331.06565, 465.06540	Negative

**Table 4:** Compounds identified in the acetone fraction QIA11 by HR-LCMS.

Compound name	Molecular structure	Retention time (min)	Found mass (*m/z*)	*m/z* values for fragment ions	Ionisation mode
Gallic acid (C_7_H_6_O_5_)	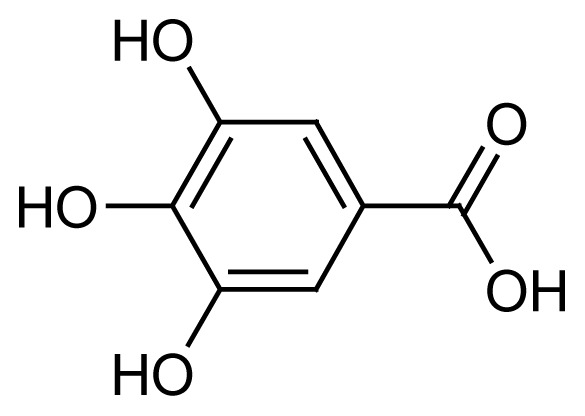 1	10.15	171.0283	68.95251, 115.05408, 169.07555	Negative
1,3,6-tris-o-(3,4,5-trihydroxybenzoyl)-beta-d-glucose (C_27_H_24_O_18_)	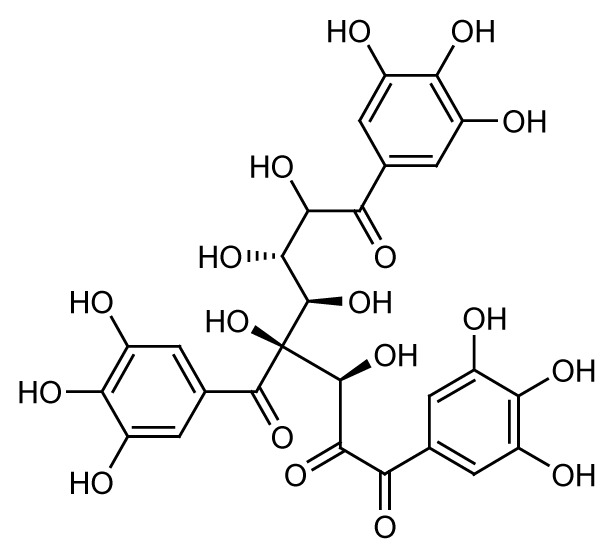 3	13.34	635.0865	271.04473, 169.01283, 313.05531	Negative
1-O,6-O-digalloyl-beta-D-glucose (C_20_H_20_O_14_)	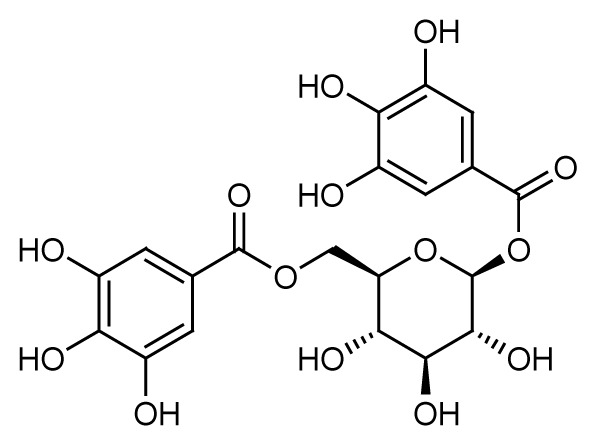	11.71	483.0761	271.04473, 169.01276, 211.02335	Negative

## References

[b1-tlsr-35-2-167] Abdullah AR, Hapidin H, Abdullah H (2017). Phytochemical analysis of *Quercus infectoria* galls extracts using FTIR, LC-MS and MS/MS analysis. Research Journal of Biotechnology.

[b2-tlsr-35-2-167] Alseekh S, Aharoni A, Brotman Y, Contrepois K, D’Auria J, Ewald J, Ewald JC (2021). Mass spectrometry-based metabolomics: A guide for annotation, quantification and best reporting practices. Nature Methods.

[b3-tlsr-35-2-167] Abdul Haque AS, Wasim A, Khan MR, Hasan A (2016). Ethnopharmacology of *Quercus infectoria* galls: A review. Hippocratic Journal of Unani Medicine.

[b4-tlsr-35-2-167] Arya A, Foko LPK, Chaudhry S, Sharma A, Singh V (2021). Artemisinin-based combination therapy (ACT) and drug resistance molecular markers: A systematic review of clinical studies from two malaria endemic regions – India and Sub-Saharan Africa. International Journal for Parasitology: Drugs and Drug Resistance.

[b5-tlsr-35-2-167] Atanasov AG, Waltenberger B, Pferschy-Wenzig EM, Linder T, Wawrosch C, Uhrin P, Temml V (2015). Discovery and resupply of pharmacologically active plant-derived natural products: A review. Biotechnology Advances.

[b6-tlsr-35-2-167] Basri DF, Tan LS, Shafiei Z, Zin NM (2012). *In vitro* antibacterial activity of galls of *Quercus infectoria* Olivier against oral pathogens. Evidence-Based Complementary and Alternative Medicine.

[b7-tlsr-35-2-167] Berthi W, González A, Rios A, Blair S, Cogollo A, Pabon A (2018). Anti-plasmodial effect of plant extracts from *Picrolemma huberi* and *Picramnia latifolia*. Malaria Journal.

[b8-tlsr-35-2-167] Blades AT, Ikonomou MG, Kebarle P (1991). Mechanism of electrospray mass spectrometry. electrospray as an electrolysis cell. Analytical Chemistry.

[b9-tlsr-35-2-167] Che CT, Zhang H (2019). Plant natural products for human health. International Journal of Molecular Sciences.

[b10-tlsr-35-2-167] Dash GK, Ansari MT, Sami F, Majeed S (2016). Proximate analysis and quantitative estimation of gallic acid in *Quercus infectoria* Oliv. galls by HPTLC. International Journal of Pharmaceutical Sciences and Research.

[b11-tlsr-35-2-167] Dworkin JP, Gargaud M, Amils R, Quintanilla JC, Cleaves (Jim) HJ, Irvine WM, Pinti DL, Viso M (2011). Chromatographic co-elution. Encyclopedia of astrobiology.

[b12-tlsr-35-2-167] Ekasari W, Fatmawati D, Khoiriah SM, Baqiuddin WA, Nisa HQ, Maharupini AAS, Wahyuni TS, Oktarina RD, Suhartono E, Sahu RK (2022). Antimalarial activity of extract and fractions of *Sauropus androgynus* (L.) Merr. Scientifica (Cairo).

[b13-tlsr-35-2-167] Fatima S, Farooqi AHA, Kumar R, Kumar TRS, Khanuja SPS (2001). Antibacterial activity possessed by medicinal plants used in tooth powders. Journal of Medicinal and Aromatic Plant Sciences.

[b14-tlsr-35-2-167] Ferguson NM (2018). Challenges and opportunities in controlling mosquito-borne infections. Nature.

[b15-tlsr-35-2-167] Ferrer Amate C, Unterluggauer H, Fischer RJ, Fernández-Alba AR, Masselter S (2010). Development and validation of a LC–MS/MS method for the simultaneous determination of aflatoxins, dyes and pesticides in spices. Analytical and Bioanalytical Chemistry.

[b16-tlsr-35-2-167] Goli AH, Barzegar M, Sahari MA (2005). Antioxidant activity and total phenolic compounds of pistachio (*Pistachia vera*) hull extracts. Food Chemistry.

[b17-tlsr-35-2-167] Hamid H, Kaur G, Abdullah ST, Ali M, Athar M, Alam MS (2005). Two new compounds from the galls of *Quercus infectoria* with nitric oxide and superoxide inhibiting ability. Pharmaceutical Biology.

[b18-tlsr-35-2-167] Harborne JB (1998). Phytochemical methods.

[b19-tlsr-35-2-167] Ibrahim N, Abu-Bakar N (2019). Measurement of pH of the digestive vacuole isolated from the plasmodium falciparum-infected erythrocyte by digitonin permeabilization. International Journal of Pharmaceutical Sciences and Research.

[b20-tlsr-35-2-167] Imtiyaz S, Ali SJ, Tariq M, Chaudhary SS, Aslam M (2013). Oak galls: The medicinal balls. Journal of Pharmaceutical and Scientific Innovation.

[b21-tlsr-35-2-167] Iylia Arina MZ, Harisun Y (2019). Effect of extraction temperatures on tannin content and antioxidant activity of *Quercus Infectoria* (manjakani). Biocatalysis and Agricultural Biotechnology.

[b22-tlsr-35-2-167] Jonville MC, Kodja H, Humeau L, Fournel J, De Mol P, Cao M, Angenot L, Frédérich M (2008). Screening of medicinal plants from Reunion Island for antimalarial andcytotoxic activity. Journal of Ethnopharmacology.

[b23-tlsr-35-2-167] Joshi DD, Joshi DD (2012). UV–vis spectroscopy: Herbal drugs and fingerprints. Herbal drugs and fingerprints: Evidence based herbal drugs.

[b24-tlsr-35-2-167] Kamarudin NA, Nik Salleh NNH, Tan SC (2021a). Gallotannin-enriched fraction from *Quercus infectoria* galls as an antioxidant and inhibitory agent against human glioblastoma multiforme. Plants.

[b25-tlsr-35-2-167] Kamarudin NA, Muhamad N, Nik Salleh NNH, Tan SC (2021b). Impact of solvent selection on phytochemical content, recovery of tannin and antioxidant activity of *Quercus infectoria* galls. Pharmacognosy Journal.

[b26-tlsr-35-2-167] Kanu AB (2021). Recent developments in sample preparation techniques combined with high-performance liquid chromatography: A critical review. Journal of Chromatography A.

[b27-tlsr-35-2-167] Kebarle P, Verkerk UH, Cole RB (2010). On the mechanism of electrospray ionization mass spectrometry (ESIMS). Electrospray and MALDI mass spectrometry: Fundamentals, instrumentation, practicalities, and biological applications.

[b28-tlsr-35-2-167] Kheirandish F, Delfan B, Mahmoudvand H, Moradi N, Ezatpour B, Ebrahimzadeh F, Rashidipour M (2016). Antileishmanial, antioxidant, and cytotoxic activities of *Quercus infectoria* Olivier extract. Biomedicine and Pharmacotherapy.

[b29-tlsr-35-2-167] Kumar N, Goel N (2019). Phenolic acids: Natural versatile molecules with promising therapeutic applications. Biotechnology Reports (Amsterdam, Netherlands).

[b30-tlsr-35-2-167] Ma S, Qin H, Jiang M, Wang J, Wang W, Guo G, Zhou L, Chen W, Han B (2020). Identification and comparison of tannins in gall of *Rhus chinensis* Mill. and gall of *Quercus infectoria* Oliv. by high-performance liquid chromatography-electrospray mass spectrometry. Journal of Chromatographic Science.

[b31-tlsr-35-2-167] Mamede L, Ledoux A, Jansen O, Frédérich M (2020). Natural phenolic compounds and derivatives as potential antimalarial agents. Planta Medica.

[b32-tlsr-35-2-167] Mazid M, Khan TA, Mohammad F (2011). Role of secondary metabolites in defense mechanisms of plants. Biology and Medicine.

[b33-tlsr-35-2-167] Mohd-Zamri NH, Sinin NJ, Abu-Bakar N (2017). Preparation and *in vitro* characterization *o*f resealed erythrocytes containing Tmr-Dextran for determination of hemoglobin uptake and transfer by the malaria parasite. International Journal of Pharmaceutical Sciences and Research.

[b34-tlsr-35-2-167] Morales D (2021). Oak trees (Quercus spp.) as a source of extracts with biological activities: A narrative review. Trends in Food Science and Technology.

[b35-tlsr-35-2-167] Nakalembe L, Kasolo JN, Nyatia E, Lubega A, Bbosa GS (2019). Analgesic and anti-inflammatory activity of total crude leaf extract of *Phytolacca dodecandra* in Wistar albino rats. Neuroscience and Medicine.

[b36-tlsr-35-2-167] Newman DJ, Cragg GM (2020). Natural products as sources of new drugs over the nearly four decades from 01/1981 to 09/2019. Journal of Natural Products.

[b37-tlsr-35-2-167] Nigussie G, Wale M (2022). Medicinal plants used in traditional treatment of malaria in Ethiopia: A review of ethnomedicine, anti-malarial and toxicity studies. Malaria Journal.

[b38-tlsr-35-2-167] Nik Mat Zin NNI, Mohamad MN, Roslan K, Sazeli AW, Abdul Moin NI, Alias A, Zakaria Y, Abu-Bakar N (2020). In vitro antimalarial and toxicological activities of *Quercus infectoria* (Olivier) gall extracts. Malaysian Journal of Medical Sciences.

[b39-tlsr-35-2-167] Nik Mat Zin NNI, Kathap MO, Sul’ain MD, Abu Bakar N (2019). Evaluation of antimalarial and toxicological activities of methanol and water leaves extracts of *Piper sarmentosum*. Asian Journal of Medicine and Biomedicine.

[b40-tlsr-35-2-167] Nik Mat Zin NNI, Wan Mohd Rahimi WNA, Abu Bakar N (2019). A review of Quercus infectoria (Olivier) galls as a resource for anti-parasitic agents: *In vitro* and *in vivo* studies. Malaysian Journal of Medical Sciences.

[b41-tlsr-35-2-167] Núñez O, Lucci P, Taylor JC (2014). Applications and uses of formic acid in liquid chromatography-mass spectrometry analysis. Advances in chemical research.

[b42-tlsr-35-2-167] Ochieng CO, Midiwo JO, Owuor PO (2010). Anti-plasmodial and larvicidal effects of surface exudates of *Gardenia ternifolia* aerial parts. Research Journal of Pharmacology.

[b43-tlsr-35-2-167] Oldoni TLC, Merlin N, Bicas TC, Prasniewski A, Carpes ST, Ascari J, de Alencar SM (2021). Antihyperglycemic activity of crude extract and isolation of phenolic compounds with antioxidant activity from *Moringa oleifera* Lam. leaves grown in Southern Brazil. Food Research International.

[b44-tlsr-35-2-167] Othman L, Sleiman A, Abdel-Massih RM (2019). Antimicrobial activity of polyphenols and alkaloids in Middle Eastern plants. Frontiers in Microbiology.

[b45-tlsr-35-2-167] Ouji M, Augereau JM, Paloque L, Benoit-Vical F (2018). *Plasmodium falciparum* resistance to artemisinin-based combination therapies: A sword of damocles in the path toward malaria elimination. Parasite.

[b46-tlsr-35-2-167] Rasoanaivo P, Ratsimamanga-tjrverg S, Frappier F, Willcox M, Bodeker G, Rasoanaivo P, Addae-Kyereme J (2021). Guidelines for the nonclinical evaluation of the efficacy of traditional antimalarials. Traditional medicinal plants and malaria.

[b47-tlsr-35-2-167] Rayleigh L (1882). XX. On the equilibrium of liquid conducting masses charged with electricity. The London, Edinburgh, and Dublin Philosophical Magazine and Journal of Science.

[b48-tlsr-35-2-167] Samuelsson G (1999). Drug of natural origin a textbook of pharmacognosy.

[b49-tlsr-35-2-167] Shah GM, Abbasi AM, Khan N, Guo X, Khan MA, Hussain M, Bibi S, Nazir A, Adnan Tahir A (2014). Traditional uses of medicinal plants against malarial disease by the tribal communities of lesser Himalayas-Pakistan. Journal of Ethnopharmacology.

[b50-tlsr-35-2-167] Shrestha S, Kaushik VS, Eshwarappa RSB, Subaramaihha SR, Ramanna LM, DB (2014). Pharmacognostic studies of insect gall of *Quercus infectoria* Olivier (Fagaceae). Asian Pacific Journal of Tropical Biomedicine.

[b51-tlsr-35-2-167] Shukla R, Dubey A, Pandey V, Golhani D, Jain AP (2017). Chromophore: An utility in UV spectrophotometer. Inventi Rapid: Pharm Analysis and Quality Assurance.

[b52-tlsr-35-2-167] Snyder LR, Dolan JW (2007). High-performance gradient elution: The practical application of the linear-solvent-strength model.

[b53-tlsr-35-2-167] Tahir AH, Hussain Z, Yousuf H, Fazal F, Tahir MA, Kashif M (2022). Traditional herbal medicine and its clinical relevance: A need to preserve the past for the future. Journal of Biosciences and Medicines.

[b54-tlsr-35-2-167] Tajuddeen N, Van Heerden FR (2019). Antiplasmodial natural products: An update. Malaria Journal.

[b55-tlsr-35-2-167] Tayel AA, El-Sedfy MA, Ibrahim AI, Moussa SH (2018). Application of *Quercus infectoria* extract as a natural antimicrobial agent for chicken egg decontamination. Revista Argentina de Microbiologia.

[b56-tlsr-35-2-167] Tu Y (2011). The discovery of Artemisinin (Qinghaosu) and gifts from chinese medicine. Nature Medicine.

[b57-tlsr-35-2-167] Uzor PF, Onyishi CK, Omaliko AP, Nworgu SA, Ugwu OH, Nwodo NJ (2021). Study of the antimalarial activity of the leaf extracts and fractions of *Persea americana* and *Dacryodes edulis* and their HPLC analysis. Evidence-Based Complementary and Alternative Medicine.

[b58-tlsr-35-2-167] Vink H (1972). Resolution and column efficiency in chromatography. Journal of Chromatography A.

[b59-tlsr-35-2-167] Walker JM (2009). Natural products isolation: Methods and protocols. Life Sciences.

[b60-tlsr-35-2-167] Wan Nor Amilah WAW, Mohamad AN, Noor Izani NJ, Arizam MF (2022). Antimicrobial activities of *Quercus infectoria* gall extracts: A scoping review. Journal of Herbal Medicine.

[b61-tlsr-35-2-167] WHO (2021). World malaria report 2021.

[b62-tlsr-35-2-167] Wu Z, Gao W, Phelps MA, Wu D, Miller DD, Dalton JT (2004). Favorable effects of weak acids on negative-ion electrospray ionization mass spectrometry. Analytical Chemistry.

[b63-tlsr-35-2-167] Yuan H, Ma Q, Ye L, Piao G (2016). The traditional medicine and modern medicine from natural products. Molecules.

[b64-tlsr-35-2-167] Zhang QW, Lin LG, Ye WC (2018). Techniques for extraction and isolation of natural products: A comprehensive review. Chinese Medicine.

